# Effects of light- vs. heavy-load squat training on velocity, strength, power, and total mechanical work in recreationally trained men and women

**DOI:** 10.5114/biolsport.2024.129487

**Published:** 2023-09-21

**Authors:** Carlos Valenzuela-Barrero, F. Javier Núñez-Sánchez, Irineu Loturco, Fernando Pareja-Blanco

**Affiliations:** 1 Physical Performance and Sports Research Center; 2 Dept of Sport and Informatics, Universidad Pablo de Olavide, Seville, Spain; 3 Nucleus of High Performance in Sport (NAR), São Paulo, Brazil; 4 Dept of Human Movement Sciences, Federal University of São Paulo, São Paulo, Brazil; 5 University of South Wales, Pontypridd, Wales, United Kingdom

**Keywords:** Resistance training, Training intensity, Sex, Volume load, Physical performance

## Abstract

The purpose of this study was to investigate the effects of light and heavy loads in the squat exercise on kinematics and mechanical variables in recreationally trained men and women. Twenty-two men and sixteen women were assigned to 4 groups: 40% and 80% one-repetition maximum (1RM) male (M40 and M80) and female (F40 and F80). Over 6 weeks, participants performed twice a week the full back-squat (SQ) exercise with initially equated relative volume load (Sets*Repetitions/Set*%1RM). All groups performed different amounts of work (p < 0.05), while relative work (work/1RM) only differed between load groups (p < 0.001). There was no significant Time*Sex*Load interaction. Based on the magnitude of effect sizes: M80 achieved small improvements in the SQ maximum isometric force (MIF; ES = 0.43, 95% CI [0.16, 0.81]); small gains in squat estimated 1RM strength were observed in the 80%-1RM groups (M80: 0.42 [0.18, 0.77]; F80: 0.44 [0.26, 0.76]) and the F40 group (0.42 [0.17, 0.81]); all groups made moderate to large gains in the average velocity attained against heavy loads (> 60%1RM; F40: 1.20 [0.52, 2.27]; F80: 2.20 [1.23, 3.93]; M40: 0.85 [0.29, 1.59]; M80: 1.03 [0.55, 1.77]), as well as small to moderate improvements in the average velocity against light loads (< 60%1RM; F40: 0.49[-0.24, 1.68]; F80: 1.10 [0.06, 3.16]; M40: 0.80 [0.41, 1.35]; M80: 0.93 [0.25, 1.84]). Lastly, only the F40 group showed small improvements in countermovement jump (CMJ) height (ES = 0.65 [0.14, 1.37]). In conclusion, light and heavy loads produced similar strength gains in men and women when initially equated by relative volume load, although the standardized mean differences suggest nuances depending on the sample and task.

## INTRODUCTION

Given the benefits of resistance training (RT) in the general population, it is recommended for both men and women in health guidelines [1]. The loading magnitude (intensity) is considered one of the most important variables when designing RT programmes [2]. The use of heavy over light loads is recommended when the goal is to develop muscular strength [3]. This assumption is supported by the idea of a “strength-endurance continuum” where a heavy-load low-repetition RT scheme promotes greater gains in strength performance, while a light-load high-repetition routine benefits endurance-related capacities more [3, 4]. This recommendation is mainly based on the variations observed in the one-repetition maximum (1RM) value. However, this approach may bias results towards heavier loads, underestimating the benefits potentially generated by lighter loads due to the fewer similarities between the training load and the exercise tested [3]. In this regard, it can be expected that training-induced adaptations would be greater for lifting conditions similar to those experienced during training [2]. Despite this, the use of light loads (< 50% of 1RM) can also be a suitable method for developing muscle strength and hypertrophy, with the additional advantage of reducing articular stress and requiring no specific facilities compared to heavy-load training [5].

The quantification and monitoring of training volume are also relevant factors in RT interventions [6], as they influence strength adaptations [4]. From a scientific standpoint, volume quantification is important not only when training volume is the independent variable, but also when the goal is to elucidate the effect of another variable, such as training load [7]. This control allows researchers to isolate the effects of the parameter under study (i.e., dependent variable). Among the variety of methods used to determine RT volume [6], volume load (number of repetitions × load) is prominent, especially because it is easy to implement [6] and is strongly associated with strength changes in both men and women [8]. Indeed, this parameter has been used in an attempt to match the volume accumulated with different loads [7]. However, this approach does not account for different parameters such as the body mass lifted in exercises such as squats, the total force applied, and the load displacement [6]. For these reasons, quantifying volume as the total amount of work performed (i.e., force [N] × displacement [m]) may be a suitable method for determining exercise volume, as it considers the total force produced and the load displacement [6].

Overall, there is a lack of studies comparing the effects of different RT interventions between men and women [9]. The same holds true for studies examining the effects of different training loads in both sexes simultaneously. Specifically, volume-matched RT protocols with multi-joint lower-limbs exercises, such as the squat, conducted with men, resulted in greater strength gains for heavy loads compared to light loads [4, 7, 10, 11]. However, studies including only women showed that both heavy and light loads were equally effective to improve strength performance [12–14]. When pooled results from a mixed-sex sample (both sexes together) were reported, again heavy and light loads provided substantial improvements without significant differences between them, although groups were not matched by volume load [15, 16]. It has already been suggested that researchers should consider expressing the results separately for both sexes for a more comprehensive analysis of the differences and similarities between the sexes [17]. Some studies have compared the effect of the same RT intervention on men and women without comparing different training loads within the study. In this regard, both sexes responded equally to heavy-load [18], light-load [19], and combined light- and heavy-load protocols [20]. To our knowledge, the potential interaction between sexes and training loads remains unknown. A better understanding of this interaction could provide deeper comprehension regarding the feasibility of light and heavy loads in men and women as minimum effective training doses [21]. In light of these considerations, the purpose of this study was to analyse the effects of different RT loads (40 vs. 80% 1RM) with an initially equated relative volume load on the physical performance of recreationally trained men and women.

## MATERIALS AND METHODS

### Procedures

An experimental study was developed to compare the effects of two loading magnitudes during full back-squat (SQ) training on men’s and women’s physical performance. Subjects were divided by sex, and then men and women were matched according to their estimated 1RM in the Smith machine SQ exercise and allocated following a counterbalanced sequence (AB-BA) among two load groups (40% and 80% 1RM), thus generating the following groups: females: F40 and F80; males: M40 and M80, respectively. All groups trained with, initially, the same relative volume load (sets × repetitions × %1RM) using the SQ exercise for 6 weeks, twice a week, for a total of 11 sessions. Only one session was performed in the last week, so there was a rest period of 4–5 days between the last session and the post-test, similar to the pre-test and the first session. Although the external load (kg) is usually changed daily or weekly in order to meet the principle of progressive overload, it was kept constant in this study throughout the intervention, even though it goes against that principle, and the relative load (% 1RM) that the same absolute load represents would decrease as their performance increases [2], which is why we are talking about “initially equated relative volume load”, since keeping the absolute external load constant would eventually lead to a different relative volume load for each subject, depending on their changes in performance during the study. The reason behind that decision was to simulate an environment where a pre-test is performed and a certain relative load is selected, with no modifications after that to see the different adaptations provided by those initial loads over time. Moreover, although training with the same absolute load for a long period might not be the most optimal regarding short-term adaptations, it could be beneficial, especially in this kind of population (recreationally trained subjects), in the medium and long term, since they could make the most out of each load, getting all (or at least more of) the positive adaptations that come from the use of that load before changing it. Participants were tested on two occasions, before and after the RT intervention, according to the following sequence: countermovement jump (CMJ); maximal isometric SQ test; and progressive loading SQ test. The same testing protocol was carried out during both pre- and post-training sessions in the same order. Testing and training sessions took place at the same time of day (± 2 h), under the same environmental conditions (20°C and 60% humidity).

### Participants

Twenty-two men (age: 23.8 ± 3.8 years; height: 1.78 ± 0.06 m; BM: 76.8 ± 10.2 kg; SQ estimated 1RM: 92.5 ± 15.1 kg; ratio 1RM to BM: 1.21 ± 0.20) and sixteen women (age: 23.0 ± 4.3 years; height: 1.62 ± 0.05 m; body mass (BM): 57.4 ± 9.8 kg; SQ estimated 1RM: 52.2 ± 12.9 kg; ratio 1RM to BM: 0.90 ± 0.17) were voluntarily recruited to participate in this study (RT experience = 2.2 ± 1.3 years, considered “recreational” based on Rhea’s classification [22]), most of them being sport science students from the university campus and some of them already familiar with lifting at maximum intended velocity, who were randomly assigned to groups. Male and female participants (age range 18–32 years) were required to be healthy and physically active individuals, regularly participating in sports or physical activities, with no physical impairments that could hinder their ability to engage in a RT programme or pose a risk to their health. No lower-body strength training or exhausting exercise was allowed outside of the study; nor was the use of any recovery treatment or anti-inflammatory supplement. This was controlled in an informal daily check on “how they had been doing” or “what kind of activities they had performed between sessions”, to make sure they were properly following the protocol. Subjects were informed about the experimental procedure and risks associated with the study, whereupon informed consent was signed. From the 41 participants who initially entered the study (number of participants per group: M40 = 12, M80 = 12, F40 = 9, F80 = 8), 3 subjects (2 from the M80 and 1 from the F40 group) dropped out due to discomfort (popliteus and low-back pain); the rest completed all programmed sessions and only their data were considerer for analysis. The study was approved by two hospitals’ ethics committees (0398-N-17) and conducted according to the Declaration of Helsinki.

### Testing Procedures

#### Countermovement Jump

Jump height was calculated through flight time measured with an infrared timing system (OptojumpNext, Microgate, Bolzano, Italy), with an accuracy of 0.001 s. Starting position: feet flat on the ground hip-width apart, hands on the hips, and looking ahead. Then, a triple flexion-extension (about 90° knee angle) was performed. Subjects were encouraged to jump as high as possible. During landing, legs were kept straight, and toes were pointing down when they touched the ground. Five jumps were performed with 20 s of rest between them, with the best and worst values discarded, and the average of the remaining 3 jumps was used for further analyses. This protocol was employed because the jump height was estimated throughout the flight time, which depends on the subject’s technique during the airborne phase. In this regard, slight modifications in the execution of the jump could inflate the real value, for example: performing ankle dorsiflexion increases the flight time and, therefore, estimates a higher jump height. Reliability was as follows: coefficient of variation (CV) = 1.8% and intraclass correlation coefficient (ICC) = 0.996, 95% CI [0.977, 0.998].

### Maximum Isometric Squat Test

Subjects started in an upright position, with hips and knees fully extended, stance approximately shoulder-width apart, feet flat on the platform parallel or externally rotated up to 15°, bar placed on the upper part of the trapezius, looking forward, neutral spine and a closed pronated grip. Then, they went down in a controlled manner until an approximately 90° knee angle was achieved, where two spotters helped them to rotate and hook the bar in the rack pins. The knee flexion angle was visually controlled by a researcher, the height of the bar being individualized for every subject and kept constant at pre- and post-training assessments. After the cue “ready, set, go!” (under a light pretension), subjects were verbally encouraged to push “as hard and fast as possible”, during a 4-s maximal effort. Two attempts with a 1-min rest were performed. The average value of the maximal isometric force (MIF) attained in each attempt was recorded. An 80 × 80 cm dynamometric platform (F-500, Ergotech, Murcia, Spain) was placed in the middle of the vertical projection of the Smith machine bar (Multipower Fitness Line, Peroga, Murcia, Spain). The force signal was sampled at 1,000 Hz and smooth with a 4^th^-order low-pass Butterworth filter with no phase shift and 200 Hz cut-off frequency. The CV for test-retest reliability was 9.2%, and the ICC was 0.950, 95% CI [0.905, 0.974].

### Progressive Loading Test in the Full Back-Squat Exercise

All subjects started with the empty bar (20 kg), in the same starting position as described in the previous isometric test. Participants were instructed to perform a controlled and continuous descent as low as possible, to immediately reverse the motion and ascend back to the upright position at the maximal intended velocity without jumping. The number of repetitions with each load and the load increments were individually adjusted as follows: 3 repetitions and 10 kg increments if the mean propulsive velocity (MPV) > 1.00 m · s^−1^, 2 repetitions and 5 kg if 0.80 < MPV < 1.00 m · s^−1^, 1 repetition and 2.5 kg if MPV < 0.8 m · s^−1^. When the MPV was lower than or equal to 0.50 m · s^−1^ (approximately ≥ 90% estimated 1RM) the test was concluded to ensure a proper technique and safety considering the subjects’ experience. The 1RM value was estimated from the MPV attained against the heaviest load lifted; first, the %1RM that that absolute load (kg) represents for a certain subject was estimated through the equation: Load (%1RM) = −5.961·MPV^2^ − 50.71·MPV + 117 [23]. Once the absolute load and its associated %1RM are known, the load in kg corresponding to their 100% of the 1RM can be calculated by a cross-multiplication (e.g., if 90 kg is estimated to be the 90% 1RM, the estimated 1RM would be 100 kg). A 3-min rest was allowed between sets. The fastest repetition for each load was considered for subsequent analysis. Velocity was measured through a linear velocity transducer (T-Force System, Ergotech, Murcia, Spain). The velocity signal was time-synchronized with the force signal coming from the previously mentioned force platform through specific software (T-Force System Version 3.60, Ergotech, Murcia, Spain). Vertical bar velocity was sampled at 1,000 Hz and smoothed with a fourth-order low-pass Butterworth filter with no phase shift and 10 Hz cut-off frequency. During the post-test, subjects followed the same loading scheme as in the pre-test, i.e., they lifted the same absolute loads, adding or removing loads depending on whether their performance was enhanced or worsened, that is, if they were able to lift more than or fewer loads before reaching the cut-off velocity (≤ 0.50 m · s^−1^). Once pre- and post-training testing had been carried out, in addition to the estimated 1RM load and to further investigate possible adaptations against different loading magnitudes, the absolute loads common to pre- and post-training were evaluated and divided into light loads (those loads lifted faster than 1.00 m · s^−1^ during the pre-test, i.e., loads lighter than the 60% of the pre-test estimated 1RM based on the general equation [23]), and heavy loads (loads lifted slower than 1.00 m · s^−1^ during the pre-test, i.e., loads heavier than the 60% of the pre-test estimated 1RM). After identifying the absolute loads of interest for every subject and their best repetition with each load (fastest repetition), the average MPV across all those loads considered light loads was calculated (AV > 1), as well as the average mean propulsive force (MPF; AF > 1) and the average mean propulsive power (MPP; AP > 1). The same procedure was conducted with those considered heavy loads to calculate the average MPV (AV < 1), average MPF (AF < 1), and average MPP (AP < 1). The number of repetitions across the average was calculated depending on every subject: since all subjects started with the empty bar (20 kg) to progressively warm up until their approximately 90% 1RM, the stronger the participant the higher the number of sets that were performed until the completion of the test.

### Resistance Training Procedures

Participants trained twice a week for 11 sessions using the Smith machine SQ exercise, with the same technique as described in the progressive loading SQ test. The absolute load (i.e. kg) for each subject was calculated as 40% or 80% of their estimated 1RM achieved at the pre-test. The number of sets, repetitions per set, and absolute load were kept constant in all training sessions (3 × 12 with pre-test estimated 40% 1RM or 3 × 6 with pre-test estimated 80% 1RM). Inter-set recovery was 3 minutes. All groups completed a standardized warm-up in every training session to isolate the effects of the independent variable (i.e. relative load), which was as follows: i) 5 min of jogging at a self-selected easy pace; ii) 2 × 10 squats without external loads; iii) 6-4-2 SQ repetitions with 20%, 40%, and 60% of 1RM, respectively. Feedback and encouragement were provided in every repetition, which was recorded using a force platform synchronized with a linear position transducer, from which the MPV, MPF, MPP, propulsive time under tension (TUT), and propulsive mechanical work were extracted. The latter, given that the software did not provide that variable or the propulsive distance, was calculated as follows:


$$\begin{array}{l} Propulsive\;Mechanical\;Work\;(J)\\ =Mean\;Propulsive\;Force\;(N)\\ \;\cdot Propulsive\;Distance\;(m)\\ =Mean\;Propulsive\;Force\;(N)\\ \;\cdot Mean\;Propulsive\;Velocity\;(m\cdot s^{-1})\\ \;\cdot Propulsive\;Time\;(s) \end{array}$$

The average propulsive mechanical work (W) of the training programme was used to compare the training performed during all training sessions by each group. To account for differences in absolute strength levels between subjects, the W completed by each subject was divided by their pretraining 1RM, obtaining the relative work (rW).

### Statistical Analyses

Data are presented as mean ± standard deviation (SD). Normality was examined with the Shapiro-Wilk test. Homoscedasticity was determined through Levene’s test. Training variables (i.e., number of repetitions, time under tension, average velocity, absolute and relative work) were analysed through a two-way ANOVA (Sex× Load). Post-training strength-related parameters (i.e., MIF, 1RM, average velocity, force and power, and CMJ) were analysed through a three-way ANCOVA (Time×Sex×Load) with Bonferroni’s post hoc comparisons, after adjusting for pretraining values with MIFpre/BM (for MIF analysis) and 1RMpre/BM as covariates, to make between-sex comparisons possible. Statistical analyses were performed using IBM SPSS Statistics v.26 (SPSS Inc., Chicago, IL). The significance level was accepted at *p* ≤ 0.05. Intraday test-retest reliabilities of the MIF and CMJ assessments were estimated from the pre-training session using intraclass correlation coefficients (ICC; 2-way mixed-effects, absolute agreement, multiple measurements) [24]. The coefficient of variation (CV) was calculated based on the standard error of measurement (SEM) [25]. Within-group effect sizes (ESs) were calculated using Cohen’s *d* for a paired design (repeated measures) and then corrected for small samples (degrees of freedom < 50), which is sometimes referred to as *d* unbiased or Hedges’ g [26]. ESs were interpreted based on Rhea’s classification for “recreationally trained” individuals (1–5 years of RT experience): < 0.35 “trivial”, 0.35–0.80 “small”, 0.80–1.50 “moderate”, and > 1.5 “large” [22].

## RESULTS

At pre-training, nonsignificant differences were found between load groups within the same sex for all strength-related parameters analysed (*p >* 0.05). In contrast, there were significant between-sex differences in most of them, except for their performance against the same absolute loads common to pre- and post-training (AV > 1, AV < 1, AF > 1, and AP > 1).

### Description of training performed

Training features are reported in Table 1. The F40 group accumulated significantly longer TUT compared to the F80 group (*p* < 0.001) and trained at a lower average training velocity (AV) compared to the male load-peers (*p* = 0.016). Men performed a greater volume load than women (*p* < 0.001). While the W was significantly different for each group (*p* < 0.05), the 40%-1RM groups completed higher rW than the 80%-1RM groups (*p* < 0.001), with no differences being found between sexes (F40 vs. M40 *p* = 0.423; F80 vs. M80 *p* = 0.605).

**TABLE 1:** Descriptive variables of the training performed during all training sessions.

	F40	F80	M40	M80
REP (n)	396 ± 0^†††^	190 ± 14	396 ± 0^†††^	194 ± 4
TUT (s)	288 ± 36^††^	231 ± 29	267 ± 24	260 ± 28
AV (m · s^−1^)	1.01 ± 0.13^†††^	0.63 ± 0.10	1.13 ± 0.12^#†††^	0.59 ± 0.06
VL (rep · kg)	8638 ± 1372	7937 ± 2082	13547 ± 1478^###^	15279 ± 2944^###^
W (kJ)	90 ± 22^†^	61 ± 14	151 ± 32^### ††^	120 ± 24^###^
rW (kJ · kg^−1^)	1.81 ± 0.42^†††^	1.16 ± 0.14	1.72 ± 0.23^†††^	1.22 ± 0.07

Data are mean ± SD. F40 = Female group training at 40% 1RM (n = 8); F80 = Female group training at 80% 1RM (n = 8); M40 = Male group training at 40% 1RM (n = 14); M80 = Male group training at 80% 1RM (n = 11); REP = Total number of repetitions performed in the training program; TUT = Total propulsive time under tension accumulated; AV = Average training velocity; VL = Absolute volume load; W = Total propulsive work; rW = Total propulsive work divided by the pretest 1RM for every subject.

Between-sexes differences:

# *p* ≤ 0.05;

## *p* < 0.01;

### *p* < 0.001.

Between-intensities differences:

† *p* ≤ 0.05;

†† *p* < 0.01;

††† *p* < 0.001.

### Strength-related parameters

Changes in the selected performance variables from pre- to post-training for each group are reported in Table 2 and Figure 2. A significant overall *Time* effect was observed for MIF (*p* = 0.011), AV < 1 (*p* = 0.001), AF < 1 (*p* = 0.034), AP < 1 (*p* = 0.011), and AV > 1 (*p* = 0.007) variables, for which within-group time effect post hoc analyses were performed. There were no significant Time*Sex, Time*Load, or Time*Sex*Load interactions for any parameter analysed. Based on the magnitude of the effect sizes, M80 was the only group that achieved small and significant MIF within-group improvements after the training programme (ES = 0.43, 95% CI [0.16, 0.81]; *p* = 0.002), while both 80%-1RM groups and the F40 group showed small gains in estimated 1RM strength (M80: 0.42 [0.18, 0.77]; F80: 0.44 [0.26, 0.76]); F40: 0.42 [0.17, 0.81]). All groups attained improvements in performance against heavy loads: moderate to large for AV < 1, trivial to small for AF < 1, and small to moderate for AP < 1 (Table 2). Likewise, although to a lessened degree, enhancements in performance were found against light loads: small to moderate for AV > 1, trivial to small for AF < 1, and small to moderate for AP < 1 (Table 2). Lastly, only the female groups showed at least small improvements in CMJ height, F40 having a 95% confidence interval that does not contain 0 (CMJ; F40: 0.65 [0.14, 1.37]); F80: 0.46 [-0.12, 1.21]).

**TABLE 2:** Mean values and pre- to posttraining changes for each group in the selected strength-related parameters.

	F40			F80			M40			M80		

	Pre-test	Post-test	ES ± [LL, UL]	Pre-test	Post-test	ES ± [LL, UL]	Pre-test	Post-test	ES ± [LL, UL]	Pre-test	Post-test	ES ± [LL, UL]
**MIF^t^**	674 ± 211	711 ± 224	0.16 ± [-0.09, 0.47]	807 ± 248	848 ± 224	0.16 ± [-0.29, 0.68]	978 ± 216	979 ± 182	0.01 ± [-0.30, 0.31]	1096 ± 224	1192 ± 205^*^	0.43 ± [0.16, 0.81]

**1RM**	51 ± 13	56 ± 10	0.42 ± [0.17, 0.81]	53 ± 14	60 ± 14	0.44 ± [0.26, 0.76]	88 ± 10	91 ± 16	0.23 ± [-0.19, 0.70]	99 ± 18	108 ± 24	0.42 ± [0.18, 0.77]

**AV < 1^ttt^**	0.67 ± 0.04	0.82 ± 0.17^*^	1.20 ± [0.52, 2.27]	0.70 ± 0.05	0.89 ± 0.10^**^	2.22 ± [1.23, 3.93]	0.73 ± 0.09	0.84 ± 0.16^***^	0.85 ± [0.29, 1.59]	0.72 ± 0.06	0.82 ± 0.13^***^	1.03 ± [0.55, 1.77]

**AF < 1^t^**	425 ± 133	486 ± 116^**^	0.46 ± [0.23, 0.84]	419 ± 108	476 ± 124^**^	0.46 ± [0.29, 0.79]	730 ± 92	786 ± 114^***^	0.53 ± [0.29, 0.88]	828 ± 176	880 ± 183^***^	0.28 ± [0.10, 0.54]

**AP < 1^t^**	260 ± 69	351 ± 44^*^	1.49 ± [0.75, 2.72]	271 ± 73	372 ± 78^*^	1.28 ± [0.79, 2.18]	487 ± 81	606 ± 159^***^	0.91 ± [0.44, 1.58]	553 ± 135	668 ± 178^***^	0.70 ± [0.36, 1.23]

**AV > 1^tt^**	1.17 ± 0.13	1.25 ± 0.16	0.49 ± [-0.24, 1.68]	1.16 ± 0.09	1.26 ± 0.07	1.10 ± [0.06, 3.16]	1.21 ± 0.08	1.34 ± 0.21^**^	0.80 ± [0.41, 1.35]	1.21 ± 0.09	1.33 ± 0.14^***^	0.93 ± [0.25, 1.84]

**AF > 1**	366 ± 59	398 ± 55	0.48 ± [0.28, 1.10]	365 ± 59	386 ± 88	0.25 ± [-0.09, 0.80]	545 ± 80	581 ± 85	0.42 ± [0.09, 0.84]	616 ± 164	656 ± 1668	0.23 ± [0.11, 0.41]

**AP > 1**	365 ± 86	420 ± 100	0.52 ± [0.17, 1.33]	335 ± 65	395 ± 77	0.73 ± [0.34, 1.77]	539 ± 95	626 ± 130	0.73 ± [0.23, 1.39]	625 ± 171	703 ± 183	0.42 ± [0.18, 0.76]

**CMJ**	23.6 ± 4.3	26.6 ± 4.4	0.65 ± [0.14, 1.37]	25.0 ± 3.0	26.3 ± 2.3	0.46 ± [-0.12, 1.21]	33.0 ± 3.4	33.9 ± 4.7	0.20 ± [-0.06, 0.51]	36.2 ± 7.1	37.5 ± 5.4	0.20 ± [-0.12, 0.57]

Data = mean ± SD. F40 = Female group training at 40% 1RM; F80 = Female group training at 80% 1RM; M40 = Male group training at 40% 1RM; M80 = Male group training at 80% 1RM; ES = Effect size; LL = Lower limit 95% Confidence Interval; UL = Upper limit 95% Confidence Interval; MIF = Maximum isometric force attained at 90° squat (N); 1RM = estimated one-repetition maximum in full-back squat (kg); AV < 1/AF < 1/AP < 1 = Average mean propulsive velocity (m · s^−1^) / force (N) / power (W) attained against absolute loads common to pre- and posttraining that were moved slower than 1 m · s^−1^. AV > 1/AF > 1/ AP > 1 = Average mean propulsive velocity (m · s^−1^) / force (N) / power (W) attained against absolute loads common to pre- and post- training that were moved faster than 1 m · s^−1^ at pre-training; CMJ = Countermovement jump height (cm). Mean Effect of time:

“t” *p* ≤ 0.05;

“tt” *p* < 0.01;

“ttt” *p* < 0.001.

Within-group time effect:

* *p* ≤ 0.05;

** *p* < 0.01;

*** *p* < 0.001.

## DISCUSSION

The present study analysed the effects of different loading magnitudes (40% vs. 80% 1RM) with initially equated relative volume load on the physical performance of recreationally trained men and women. The main findings were as follows: a) the adaptations provided by light and heavy loads were similar between men and women; b) both light and heavy loads produced small to moderate improvements in most variables studied; c) men accumulated higher total mechanical work than women; however, differences between sexes disappeared when work values were normalized to their estimated 1RM.

Although no statistically significant differences were found between sexes (men vs. women) or load groups (40% 1RM vs. 80% 1RM), the study of the within-group standardized mean differences may provide a deeper insight into the possible adaptations coming from the use of different loading magnitudes on each sex. The similarities between task requirements and training load may explain the findings observed in MIF and estimated 1RM strength in men since the 80% 1RM intervention was the only male group that obtained non-trivial gains in those maximum or near maximum force expressions. The greater effect observed in MIF for M80 in comparison to the other groups, together with being the only group where a significant within-group time effect was found (*p* = 0.002), agrees with the current literature, since men have been shown to benefit from heavy-load training [27–29], but this effect has not been observed in women [30]. Additionally, when men and women trained combining both light and heavy loads (i.e. from body weight up to 85% 1RM), greater improvements were found in men [20]. Although sex differences in MIF currently continue to be unexplained, it is plausible to consider that increases in muscle size may play a substantial role that could benefit men [20]. Unfortunately, hypertrophic responses were not evaluated in the present study. These results suggest that heavy-load training may benefit MIF production in men while the effect on women is trivial. In contrast, both the M80 and F80 groups obtained similar gains in estimated 1RM strength. In this regard, our results agree with previous studies conducted with men and matched by volume load, which showed that heavy-load training was effective to induce gains in 1RM strength [4, 7, 10, 11]. As mentioned above, the use of higher training loads may have more similar requirements and thus be more transferable to tasks with maximum or near-maximum force requirements, evoking greater adaptations to the 1RM test [31], and possibly induce larger increases in maximal neural activation and muscle thickness [29]. In agreement with the current literature, similar improvements in women’s 1RM have been observed with both light- and heavy-load RT interventions [12–14], although the use of heavier loads could be more beneficial in older adults [30]. The reason why a light-load programme was not a sufficient stimulus to achieve at least small gains in estimated 1RM strength in men, as opposed to women, may lie in the extension of the braking phase. As shown in Table 1, the average training velocity of the M40 group was significantly higher than the F40 group (*p* = 0.016). Thus, male subjects had to actively brake the movement to avoid jumping to a greater extent than their load-paired female subjects [32], affecting the potential transfer to a maximum or near-maximum test, where subjects are required to apply force throughout the entire concentric phase [23].

Force, velocity, and power outputs against light and heavy loads showed a similar pattern among them, although the within-group effect sizes in velocity values tended to be higher. As previously reported, both male groups achieved non-trivial improvements with no differences between them in the velocity-load [28, 29, 33], and power-load [33] relationships. However, the trivial improvements for the M80 group found in the present study regarding the force-load relationship contradict those seen in the literature [29], possibly because of the selected variable (mean force instead of peak force). However, it should be mentioned that the mechanisms potentially responsible for these adaptations were shown to differ depending on training intensity [29]. In concordance with a previous study [13], female groups enhanced their performance against both light (small to moderate) and heavy (moderate to large) loads, again with no differences between groups. In mixed-sample research with heavy loads, men increased power output significantly more against light-moderate loads compared to women [18], something that cannot be drawn from our results. Taken together, data from the present study suggest that both light and heavy RT loads could be equally suitable for improving performance against light and heavy loads in both men and women.

While no significant differences were found between groups regarding CMJ height, F40 was the only group that showed small improvements with a 95% confidence interval that did not contain 0. Studies with men have found light and heavy RT loads to equally improve CMJ performance; however, the “heavy-load groups” always completed a greater volume load using alternatively the squat, the jump squat, or a combination of both exercises, whereas the “light-load groups” performed only jump squats, without stopping the movement at the end of the concentric phase [27, 29, 34]. Similar results have been obtained with women [12, 13]. It is unknown how or to what extent these differences in volume load could have affected the outcomes. Additionally, as in our study, no between-sex differences were found when a light-load RT was applied to a mixed sample [19]. In a similar manner as presented in the discussion of estimated 1RM results, even considering that lighter loads could be more suitable to improve jumping performance, the higher training velocity (AV) attained by the M40 group has certainly required a longer braking phase to avoid jumping [32], which may have reduced the potential benefits of this load, since jumping was not allowed in our study. This fact was attenuated in the F40 group given its slower AV (Table 1).

**FIG. 1:**
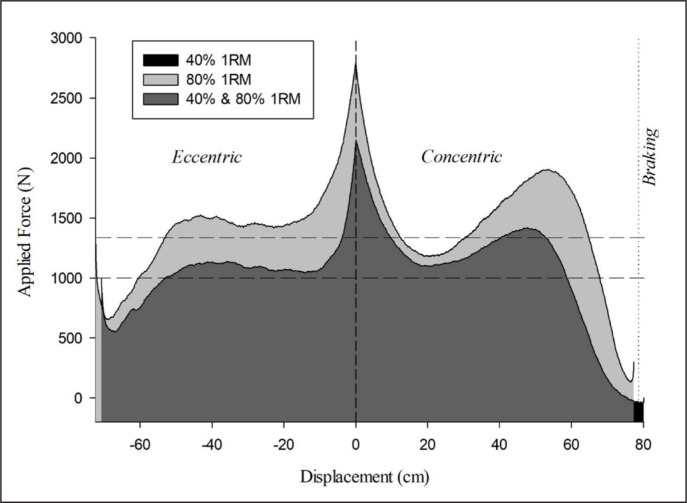
Forces comparison when lifting the 40% and 80% 1RM for a representative subject. The two curves denote the applied force, as a function of displacement, with a mean value of 1116 N for the 40% 1RM load and 1429 N for the 80% 1RM load. The two horizontal dashed lines represent the system weight (external load + 88% body mass) for each load. The vertical difference between the curves and their corresponding dashed line depicts the net force (mean value 40% 1RM load: 115 N; 80% 1RM load: 59 N). The areas under the curves show the work performed to lift the respective loads (40% 1RM load: 1570 J; 80% 1RM load 2128 J). The dark grey area (40% 1RM) represents 74% of the light grey one (80% 1RM).

Although both load interventions were initially equalized in terms of relative volume load (Sets × Repetitions/Set × %1RM), the absolute volume load was different between sexes, and the 40%-1RM groups performed more work than the 80%-1RM groups (both in absolute and relative terms). This finding is in agreement with previous studies [27, 30, 34] and indicates that volume load and actual work should not be equated at the same time. Since volume load does not account for the total force applied [6], when this force is measured during one repetition at maximal voluntary velocity, it can be observed that the force applied at 40% 1RM is greater than half that required to lift the resistance equivalent to 80% 1RM, especially when body mass is included (curves in Figure 1). Indeed, Figure 1 shows that the area under the force-distance curve (mechanical work) at 40% 1RM was 74% of the area at 80% 1RM. For this reason, 2 repetitions at 40% 1RM require performing greater work than 1 repetition at 80% 1RM. Accordingly, although lifting 80% 1RM requires a higher force to be applied than when lifting 40% 1RM (curves in Figure 1), the net force (i.e., the difference between the applied force and the force that the weight represents) is higher when lifting the 40% 1RM load (vertical difference between the force curves and their corresponding horizontal lines in Figure 1). This fact explains why the 40% 1RM load can be lifted faster than the 80% 1RM load. Given that the applied force is considerably higher than the load when lifting lighter loads compared to heavier loads, there is an underestimation of the actual work performed, especially with light loads, when equated by volume load. Furthermore, in the present study, men performed greater total work than women; however, when total work values were relativized by the subjects’ estimated 1RM, these differences disappeared (Table 1). Hence, sex differences in total mechanical work seem to be due to differences in maximum strength levels.

**FIG. 2:**
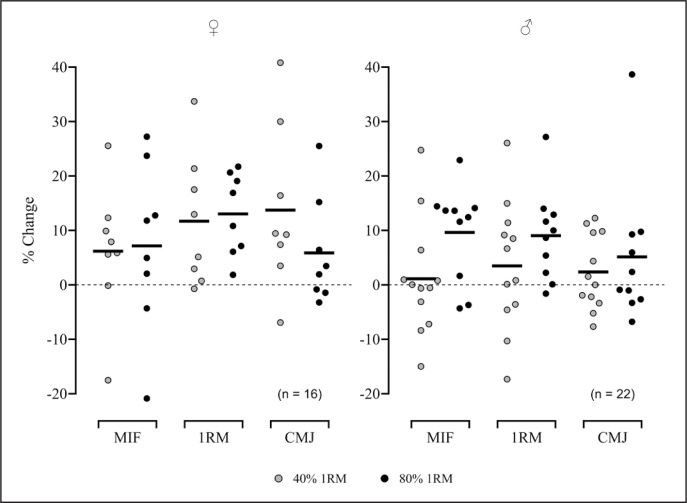
Percent change pre- to post- training for every subject in three selected strength-related parameters: ♀ = Women; ♂ = Men; MIF = Maximum isometric force attained at 90° squat (N); 1RM = estimated one-repetition maximum in full-back squat (kg); CMJ = Countermovement jump height (cm).

A drawback of the present study is its statistical power due to the sample size [35]. For instance, the observed power to detect a significant (*p* ≤ 0.05) Time*Sex*Load interaction was approximately 25%, which falls below the recommended 80%. These circumstances justify the use of additional analyses such as the effect sizes together with their confidence intervals to provide a deeper understanding of the effects that are taking place [35]. Subjects were recreationally trained so the results are not generalizable to other populations. On the other hand, the relative volume load was not equated during the entire duration of the study since the external load was kept constant, and for every subject that initial load would represent a different percent of their 1RM by the end of the study. Additionally, the effect of the variable *“Load”* has been investigated in isolation for just one exercise (full back-squat) in the present study; thus, the results may differ in an RT programme where multiple exercises are performed for the same muscle groups or movement patterns.

Lastly, some of the results obtained in the 40% 1RM male group could be attributed to the effect of the braking phase when a light load is intended to be lifted as fast as possible without jumping in the squat exercise. For all the aforementioned reasons, it would be interesting to further investigate how different loading magnitudes influence men’s and women’s performance in the future, addressing the limitations of the present study by using a larger sample size to confirm the possible differences in strength adaptations suggested by the within-group standardized mean differences that resulted from the use of different training loads in men and women, as well as recruiting participants with different training experiences, keeping the relative load constant, and/or performing multiple exercises for the same muscle group, including those where there is no braking phase, such as in ballistic exercises (e.g. jump squats at 40% and 80% 1RM).

## CONCLUSIONS

In conclusion, light and heavy loads produced similar strength gains in men and women when initially equated by relative volume load. However, based on standardized mean differences, heavier loads seem to provide more maximum and near-maximum force adaptations (MIF and estimated 1RM) in men, while women could equally benefit from either low or high loading magnitudes to improve near-maximum dynamic force production (estimated 1RM). Although light loads (40% 1RM) may be more applicable to optimize force application in tasks using only the body weight as workload (e.g. jump tasks in the F40 group), this “potential benefit” may be compromised in stronger subjects (i.e. M40 group), who tend to decelerate more aggressively at the end of the lift. It is important to emphasize that greater mechanical work is performed when training with lighter loads and equalizing by relative volume load; hence, more fatigue may be induced, which might delay a complete recovery from the RT session [36].

The decision to keep the external load constant recreates a situation where the absolute training load (kg) is based on a pre-test and it remains unchanged for several weeks until another test is conducted with no adjustment during the training period, as is the case when no rate of perceived exertion RPE, jump assessments, velocity measurements or any other methods are used to adjust the training load. In this way, we could observe the adaptations caused by those initial relative loads, something that has not been thoroughly studied to the best of our knowledge. Even though it might not be the most optimal way to improve short-term performance, training with the 40% 1RM, or rather what initially was the 40% 1RM, still produced some positive adaptations, even when at the end of the training period it probably meant a lighter load. The same held true for the initial 80% 1RM load, some groups achieving greater effect sizes and then more positive adaptations in tasks with maximum or near-maximum force production requirements (maximum isometric force and estimated 1RM). These findings help to provide some insight regarding the viability and progression of certain loads when focusing on making the most out of each load, especially important when looking at the mid- and long-term development of this kind of population.
